# Differences in Hospitals’ Workplace Violence Incident Reporting Practices: A Mixed Methods Study

**DOI:** 10.1177/15271544221088248

**Published:** 2022-03-23

**Authors:** Rachel Odes, Susan Chapman, Sara Ackerman, Robert Harrison, OiSaeng Hong

**Affiliations:** 1 Postdoctoral Fellow, National Clinician Scholars Program, 8785 University of California San Francisco, 490 Illinois St., Floor 7, San Francisco, CA 94158; 2 Professor, Department of Social and Behavioral Sciences, School of Nursing, 8785 University of California San Francisco, 490 Illinois Street, San Francisco CA 94158; 3 Associate Professor, Department of Social and Behavioral Sciences, School of Nursing, 8785 University of California San Francisco, 490 Illinois Street, San Francisco CA 94158; 4 Sr Physician Diplomate, Department of Occupational and Environmental Medicine, School of Medicine, 8785 University of California San Francisco, 2330 Post Street, San Francisco CA 94115; 5 Professor, Community Health Systems, School of Nursing, 8785 University of California San Francisco, 2 Koret Way, San Francisco CA 94143

**Keywords:** workplace violence, hospitals, risk management, incident reporting, nursing, california, data collection

## Abstract

Workplace violence (WV) is a significant and growing problem for health care workers. Increased recognition of the need for improved protections has led to policy initiatives at the state and federal levels, including national Joint Commission requirements that went into effect January 2022. California’s WV prevention legislation was phased in during 2017-2018 and requires hospitals to use a new incident reporting system, the Workplace Violent Incident Reporting System (WVIRS) for Hospitals. We analyzed WVIRS data collected during the first three years of its implementation, July 1, 2017 – June 30, 2020. In addition, we collected qualitative data from six California hospitals/hospital systems during 2019-2020 to better understand reporting practices. Over the three-year period, the 413 hospitals using the WVIRS reported between zero and six incidents per staffed bed. Sixteen hospitals (3.9%) reported two or more incidents per staffed bed while the rest reported fewer than two incidents. Qualitative analysis identified that reporting procedures vary considerably among hospitals. Several organizations rely on workers to complete incident reports electronically while others assign managers or security personnel to data collection. Some hospitals appear to report only those incidents involving physical harm to the worker. Regulatory guidance for reporting practices and hospitals’ commitment to thorough data collection may improve consistency. As hospitals throughout the U.S. consider practice changes to comply with new WV standards, those engaged in implementation efforts should look closely at reporting practices. Greater consistency in reporting across facilities can help to build evidence for best practices and lead to safety improvements.

Workplace violence (WV) in health care is a significant issue with implications for workers, patients, and hospitals. The Occupational Safety and Health Administration (OSHA) defines WV as “any act or threat of physical violence, harassment, intimidation, or other threatening disruptive behavior that occurs at the work site” (Occupational Safety and Health Administration, n.d.). Research conducted by the Emergency Nurses Association reporting on over 3,000 U.S. emergency department nurses’ experiences of WV found that 25% had experienced more than 20 incidents of physical violence over the prior three years and a third had considered leaving the profession or department as a result (Gacki-Smith *et al*., 2009). For nurses working in the critical care setting, WV is also widespread, with 47% reporting an incident of physical abuse during the previous year (Ulrich *et al*., 2019). A meta-analysis including findings from 253 international studies found that the prevalence of physical violence for healthcare workers was 24% during the past year’s work (Liu *et al*., 2019).

The impact of these events can be consequential for workers’ self-perception and competence, leading to what qualitative researchers described as “hypervigilance,” “generalized fear,” and a disengagement from caring during daily work activities (Forte *et al*., 2017). These effects are compounded by the increased strain on the health care workforce since the onset of the COVID-19 pandemic. Since the pandemic began, surveys of providers in emergency departments and of those providing care to patients with COVID-19 have shown that WV has increased, reflecting the intensity of the working conditions in hospitals around the world (Byon *et al*., 2021; McGuire *et al*., 2021).

In April 2021, the U.S. House of Representatives passed House Resolution 1195, the Workplace Violence Prevention for Health Care and Social Service Workers Act, with bipartisan support (United States Congress, 2021). This new law would require covered employers to submit an annual summary of WV incidents to the Secretary of Labor in addition to training staff in WV prevention. Further, The Joint Commission, the largest and most influential hospital-accreditation organization in the U.S., now requires participating organizations to demonstrate that they have taken steps to proactively analyze their work environments, train staff, and document incidents of WV towards staff (The Joint Commission, 2021).

In California, the legislature took action to address WV in health care in 2014 by passing Senate Bill 1299, with requirements phased in during 2017-18 (State of California Legislative Counsel, 2014). In general, the requirements from California’s Occupational Safety and Health Administration (CalOSHA) are similar to the proposed federal law. One difference is in incident reporting: California’s psychiatric and general acute care hospitals must submit reports describing incidents where force was used within 72 h of the occurrence through a web-based Workplace Violent Incident Reporting System (WVIRS) for Hospitals. A summary of incidents reported through the first year of this system and an evaluation of hospital location, size, and ownership characteristics as risk factors was previously reported (Odes *et al*., 2020).

Consistency and accuracy in WV reporting practices in health care facilities has been a long-established problem for researchers investigating prevalence and intervention efficacy (Arnetz *et al*., 2015; Bensley *et al*., 1997; Findorff *et al*., 2005; Jacobsen, 2016; Kvas & Seljak, 2014; Morphet *et al*., 2019; Peek-Asa *et al*., 2001). In particular, nurses and other health care providers have been shown to underreport incidents of violence because they feel it is unlikely to improve safety or violence is simply “part of the job” (Benson *et al*., 2003; Morphet *et al*., 2019). At the same time, organizational behavior and leadership characteristics can influence both violence and reporting practices (Findorff *et al*., 2004; Gacki-Smith *et al*., 2009). While prior research has examined these characteristics separately, no investigations have utilized a dataset comparable to what exists in California to compare hospital reporting practices.

The WVIRS provides a new way to estimate the incidence of WV in hospitals in a large, diverse state. In addition, data reported through this new channel may contribute to an understanding of risk factors and organizational responses to incidents of violence, leading to more effective mitigation strategies. In utilizing this new database, it is crucial to consider how differences in organizational practices related to the reporting system may contribute to what appears in the final record. Hospital leaders’ approaches to meeting California’s reporting requirements have yet to be fully described. The purpose of this study is to explore differences in these practices and link them to what appears on the WVIRS.

## Theoretical Approach

The approach to describing and measuring organizational practices in California’s hospitals used here is informed by institutional theory**.** Originating in sociology, institutional theory provides insight into how health care organizations behave in an environment with multiple, complex, and competing demands (Meyer & Rowan, 1977). The buffering process necessary to accomplish this is led by administrators who perform the activities needed to appear transparent to scrutiny from outsiders while permitting core practices to remain undisturbed. When considering hospitals’ implementation of California’s WV prevention legislation, institutional theory can help illuminate the multiple points of pressure exerted on those responsible for compliance. Recognition of these competing demands and motivations provided background to prepare for qualitative investigation. Prior to conducting interviews and site visits, researchers discussed the potential challenges posed to organizational leadership in full and accurate WV reporting, then reviewed these themes during the process of analysis. In addition, the idea that hospital leadership may not seek to fully disclose all of the true practices to outside researchers motivated the request to include frontline staff in the interview process where possible.

## Data and Methods

To better understand the relationship between what appears in the WVIRS and what is happening at the organizational level to generate these data, this study used mixed methods with an explanatory sequential design (Creswell & Plano Clark, 2017). Mixed methods can be useful for describing policy implementation because it leverages the ability of quantitative findings to describe phenomena broadly and the strengths of qualitative findings which tell the stories of individuals and their local contexts (Palinkas *et al*., 2011). In this case, quantitative findings were first analyzed and then questions seeking to better understand facilities’ reporting practices were devised by members of the research team. Qualitative investigation took a descriptive approach (Kim *et al*., 2017), seeking to simply develop better understanding of hospitals’ practices related to the new reporting requirements. Quantitative and qualitative findings were combined to describe differences in California hospitals’ reporting practices. The study was approved by the University of California San Francisco Institutional Review Board and the State of California Committee for the Protection of Human Subjects.

### Quantitative Data

Data reported through the WVIRS to CalOSHA are available to the public through the California Public Records Act. Records from July 1, 2017 – June 30, 2020, were included in this analysis. Hospitals’ ownership data were obtained from the California's Office of Statewide Health Planning and Development (OSHPD) and the California Department of Public Health (CDPH).

Two outcomes of interest were calculated for each of the hospitals included in this analysis. First, the total number of incidents reported through the WVIRS for each hospital was tabulated. Second, the total number of incidents involving a physical injury to the worker was calculated. This binary outcome was determined to include injuries reported in the WVIRS as death, amputation, asphyxiation, burns, bruising/abrasion, cut/puncture, dislocation/fracture, head injury, internal injury, open wound and sprain/strain. The categories stress/psychological impairment, injury type not listed, and injury type unknown by the hospital at this time were classified as non-physical injuries.

Although California’s regulation states that all incidents where physical force is used must be reported through the online portal, regardless of whether or not an injury is sustained (California Occupational Safety & Health Administration, 2016), there may be differing interpretations of this requirement. Calculating the percentage of each facility’s total reported incidents that involve a physical injury to the victim is one way to assess the organization’s interpretation of what needs to be reported and provides a starting point for assessing differences in practices. The WVIRS does not include an assessment of the overall severity of each reported incident. Therefore, it is difficult to determine how facilities have approached reporting more or less serious WV incidents. The differences in reporting related to physical injury is a starting point for this inquiry seeking to improve interpretability of what populates the WVIRS.

The first hospital characteristic (characteristics are treated here as predictors) included in quantitative analysis is whether its nurses are represented by a union. This variable was included because of the leading role California’s health care workers’ unions played in passing the state’s law and in pressing for continued compliance with the WV standard in hospitals. Unionization data were obtained from union websites and internet news searches. Second, each hospital was classified by its OSHPD-designated ownership category. These categories include City/County owned, District owned, State owned, Non-profit, or Investor owned. The five State owned psychiatric hospitals have been granted a variance from the requirements of the WV legislation, and due to the differences in their reporting practices, have been excluded from this analysis.

Lastly, each hospital’s number of staffed beds was determined. OSHPD tracks facilities’ total licensed and staffed beds and reports them as public data on a quarterly basis (OSHPD, 2020). Once obtained, these data were merged with WVIRS reports. For the 75 hospitals which did not have staffed beds listed, the number of licensed beds was obtained from either OSHPD or CDPH and was multiplied by the mean percentage of staffed beds/licensed beds observed in the other reporting hospitals (0.85). The proportion of licensed beds which were staffed for hospitals reporting this information ranged from 0.28–1.0 with a standard deviation of 0.2. This imputation method was used for ten facilities which are included in the mixed methods analysis described in Table 3. Sensitivity analysis was conducted for these facilities to account for the imprecision of this method; a lower bound of 0.45 percent of total licensed beds (mean −2sd) and an upper bound of 1 (or all licensed beds) was included in the final table to indicate what impact use of this method may have on results.

**Table 3.** Integrated Findings: Dimensions of Reporting with Incidents per bed and Percentage of Incidents Involving Injury.

Facility Number	Number of reporting entities included	Organizational leadership responsible for WV compliance	Personnel responsible for data collection/entry into WVIRS	Staff training: online	Staff training: in person	Total incidents per staffed bed	Percentage (%) of incidents involving staff injury
						July 2017 – June 2020	
						When multiple facilities reporting, mean value provided with 95% CI	
1	3	Law enforcement	Security collects data from staff, enters into WVIRS	None	New employees 6.5 h in person, yearly refreshers (can “test out” with written exam)	Mean: 1.34 (.19–2.01) *Sens analysis for staffed beds: 1.32–1.40*	Mean: 4.0% (0–12.7%)
2	1	Specialized WV personnel; law enforcement background	Staff enter data into incident reporting system; risk manager cleans, enters into WVIRS	All staff: 30 min annually	Behavioral health, emergency dept. and security staff: 8-h annual	1.29	19.8%
3	4	Patient care	Staff enter data into incident reporting system; risk manager cleans, enters into WVIRS	All staff: 30 min annually	varies	Mean: .32 (.08 − .56) *Sens analysis for staffed beds: .31 − .36*	Mean: 27.0% (0-68.0%)
4	4	Patient care	Staff enter data into incident reporting system; risk manager cleans, enters into WVIRS	All staff: 30 min annually	Behavioral health, emergency dept. and security staff: 8-h annual	Mean: .55 (0–1.2) *Sens analysis for staffed beds: .51 − 63*	Mean: 35.5% (11.9% − 55.1%)
5	30	WV consultant; security background	Unit manager enters data into incident reporting system; risk manager cleans, enters into WVIRS	All clinical staff: one hour annually	Provided to individual units by request	Mean: .04 (0.03–.06) *Sens analysis for staffed beds: .04–.05*	Mean: 63.7% (51.5–75.9)
6	1	Patient care	House supervisor (RN) collects data, enters into WVIRS	Emergency dept and security staff: 4-h annual	Emergency dept and security staff: 4-h annual	.04	75.0%

At the onset of qualitative data collection, preliminary analysis was conducted on the available quantitative data (2017-2019). In particular, the researchers calculated each facility’s total incidents per staffed bed, percentage of incidents involving a physical injury, and hospital unit or location with the most reported incidents. These findings provided a context for interviews and discussion with interview respondents and facilitated interpretation of data gathered during the qualitative process. While quantitative data collection was ongoing during the qualitative process, the availability of these preliminary findings to inform researchers’ approach to interviewing aligns with the explanatory sequential design.

### Qualitative Data

During 2019-2020, two interviewers conducted site visits and phone interviews with hospital representatives under the auspices of CDPH’s Occupational Health Branch (OHB). Participants were informed that site visits and interviews were for research purposes only and that findings would not be used to issue citations. The first interviewer was an experienced psychiatric nurse who was working as a contractor for CDPH for the duration of this project. The second interviewer was an industrial hygienist employed by CDPH with extensive experience in workplace safety evaluation and education. Interviews ranged from one-hour phone calls to day-long site visits, depending on facility representatives’ availability and preference.

The first interviewer’s written notes provide the primary data source, and the second interviewer provided additional data from notes and corroboration. Observations related to the worksite environment or other safety measures described were also recorded by the first interviewer. The first interviewer took notes during each meeting, then reviewed and completed the summary of each interview or site visit over the next day. This process involved both a thorough description of what was discussed, and a reflective process describing observations and summaries to capture broad impressions of the environment and discussion and aid in comparison between facilities (Creswell & Plano Clark, 2017). Notes were shared with the second interviewer and discussed to verify accuracy and completeness. Recordings were not obtained to encourage candid discussion of potentially sensitive topics.

Participating hospitals/systems represented a convenience sample of the state’s approximately 400 hospitals required to use the WVIRS. The California Hospital Association (CHA), a trade organization for the state’s hospitals, was active during the stakeholder engagement process of the WV legislation development and continued to have a working group devoted to the issue. Four of the six participating hospitals/systems volunteered to participate after receiving information about the project from the CHA’s electronic list-serve. The other two hospitals were contacted by the OHB directly based on geographical proximity to the researchers. An additional, county-funded public hospital was contacted by OHB but did not agree to participate, and another hospital which is a member of a large, non-profit health care system agreed to participate but canceled due to the start of the coronavirus pandemic in Spring 2020.

The six hospitals/systems which participated in interviews and site visits during the qualitative investigation are described in Table 2. The number of facilities represented by each interview or site visit is noted in Table 3. Participants were hospital representatives with responsibility for ensuring compliance with the WV standard. Some worked in security operations and others had risk management or patient care management responsibilities. Interviewers requested that unit-level managers and frontline staff be invited to participate; these individuals were included at participants’ discretion and were present for two interviews. In preparation for qualitative data collection, the researchers consulted the Consolidated Criteria for Reporting Qualitative Studies (COREQ) checklist to promote transparency and completeness in the data collection process (Tong *et al*., 2007).

**Table 2.** Description of Hospitals or Hospital Systems Providing Qualitative Data.

Facility Number	Interview method	Hospital/system description	Unionization	Facility houses Behavioral health unit and/or Emergency department
1	In person interview/tour	Privately owned, non-profit; rural	Nurses unionized	Substance abuse treatment unit and emergency department
2	In person interview	Acute care facility within statewide system; urban	Nurses, ancillary personnel unionized	Emergency department
3	Phone interview	Multiple facilities comprising statewide system; urban/suburban	Nurses, ancillary personnel unionized	Behavioral health units and emergency departments
4	In person interview	Acute care facilities in single city within statewide system; urban	Nurses, ancillary personnel unionized	Behavioral health unit and emergency department
5	Phone interview	Large, statewide system; varying locations	Nurses, ancillary personnel unionized	Behavioral health units and emergency departments
6	In person interview/tour	Small, non-profit facility; rural/suburban	Not unionized	Emergency department

### Qualitative Analysis

The overall approach to qualitative analysis was informed by Braun and Clarke’s (2006) description of thematic analysis. Their guidance suggests that thematic analysis provides a useful way to answer a limited set of questions through interpretation of qualitative data. The first author was responsible for conducting the thematic analysis and used Braun and Clarke’s six steps to provide structure to the development and refinement of codes and lend rigor to the process.

Thematic analysis began with the first author’s review of the interview notes and identification of practices relevant to reporting procedures within each hospital. Once the initial review was complete, the first author then collated related sections from the interview notes using a spreadsheet to visualize the number of comments on a single topic. Referring to this spreadsheet, the first author identified an initial set of key ideas including all hospital practices related to WV incident reporting. These were refined and grouped into four themes: reporting, leadership, training, and overall approach to WV prevention. While participants’ comments and experiences provided the data, it was clear that the interviewers’ prompts and interest in specific aspects of reporting practices also shaped the responses (Braun & Clarke, 2006; Vaismoradi *et al*., 2013). Sections from all the interview notes that fit into each of the identified themes were then collated together in a new spreadsheet to determine whether themes were coherent and captured a distinct topic area. Due to the use of interviewers’ notes instead of transcripts, organizing content on spreadsheets was less cumbersome than qualitative analysis software.

### Statistical and Mixed Methods Analyses

Quantitative data analysis was conducted in STATA SE/15. A 2-tailed t-test was used to compare reported incidents involving physical injury between unionized and non-unionized hospitals. Significance was set at p = <.05. Mixed methods analysis was conducted using an explanatory sequential approach (Creswell & Plano Clark, 2017). Quantitative data were collected and tabulated first, and facilities were listed in descending order based on the number of incidents per staffed bed. Then, columns summarizing the major domains of qualitative findings were used to better visualize the relationship between qualitative and quantitative findings.

## Results

### Quantitative Findings

Description of the California hospitals (n = 413) reporting through the WVIRS from July 1, 2017 – June 30, 2020, is provided in Table 1. The majority of the state’s hospitals are operated by private, non-profit entities (61%). From July 1, 2017 – June 30, 2020, a total of 27,968 incidents were reported through the WVIRS. The assailant was described as a patient in 94% of incidents and as a patient’s family member in 2% of incidents. A physical injury to the worker was reported in 32% (n = 8943) of total incidents.

**Table 1.** Description of Hospitals (N = 413) that Reported Incidents Using the WVIRS (July 2017 – June 2020).

Facility ownership^†^	Frequency	Percentage (%)
City/County	25	6%
District	37	9%
Non-profit	250	61%
Investor	101	24%
**Type of hospital**		
Psychiatric hospital	29	7%
General acute care hospital	384	93%
**Unionization status**		
Nurses unionized	237	57%
Nurses not unionized	176	43%

^†^ State hospitals’ data excluded.

**Figure 1.**
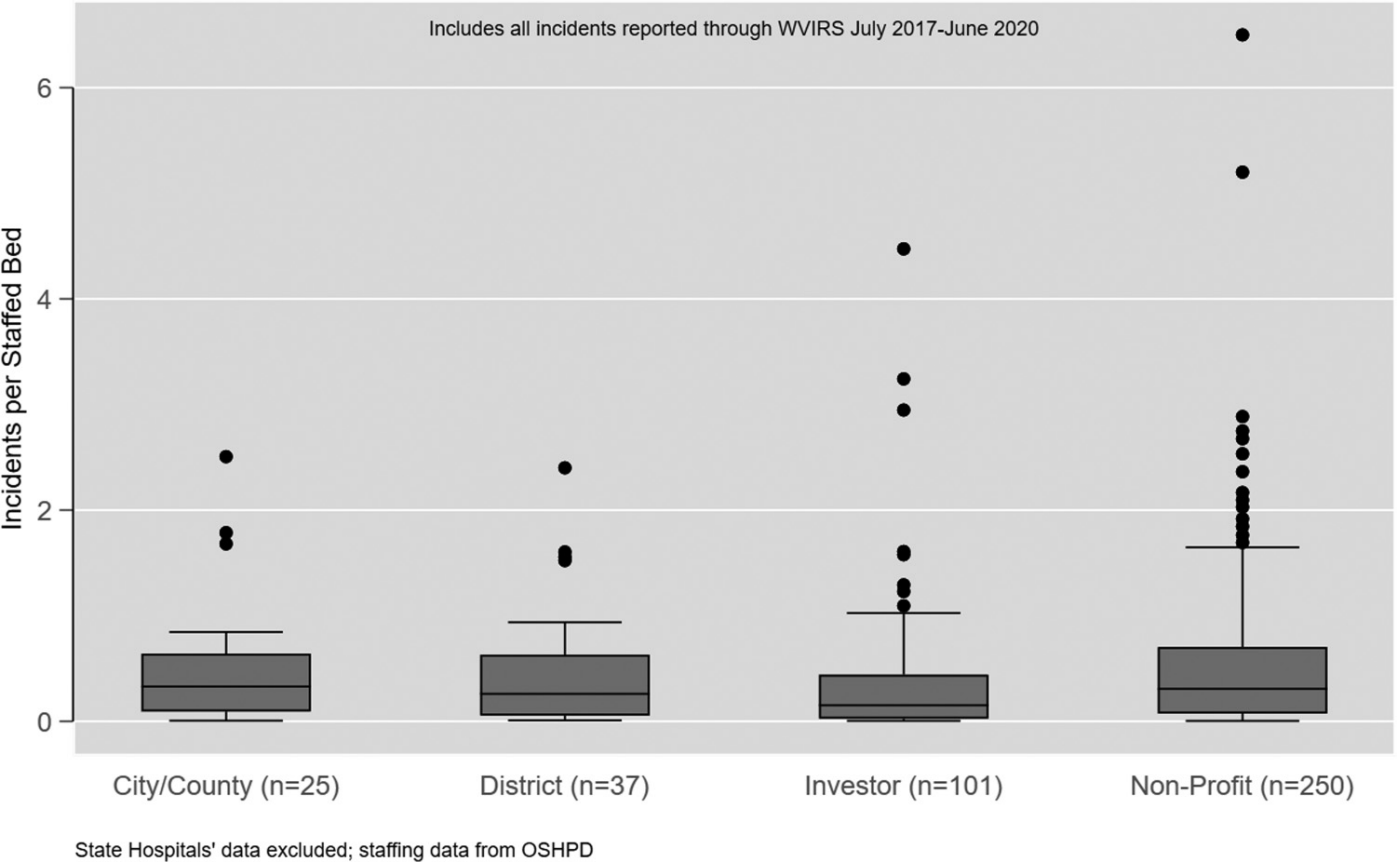
Total Incidents Per Staffed Bed (N=413 Facilities).

**Figure 2.**
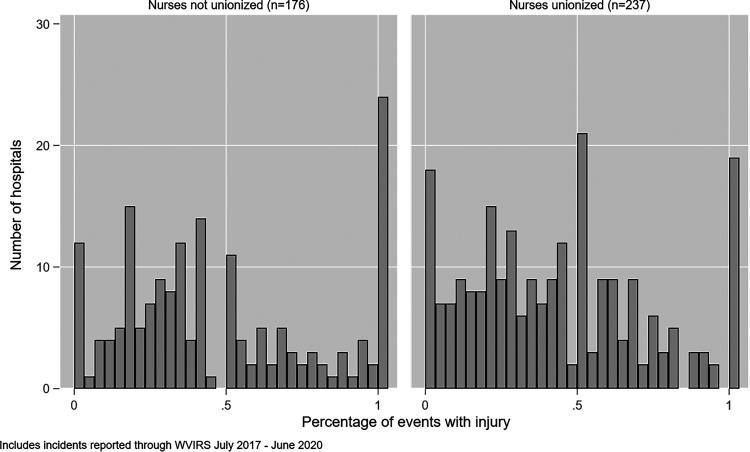
Percentage of Incidents Involving Physical Injury by Unionization (N=413 Facilities).

Reporting hospitals vary in size, with staffed beds ranging from four to 880. Therefore, to compare number of incidents between facilities it is helpful to adjust for number of staffed beds. The total reported incidents are shown in Figure 1. A total of 16 hospitals (3.9%) reported two or more incidents per staffed bed. The distributions of hospitals’ reported incidents involving a physical injury to the victim are presented in Figure 2. For hospitals with zero percent of their incidents involving injury, this can be interpreted to mean that all incidents reported did not involve physical harm to the impacted worker. There was no significant difference in the mean percentage of incidents involving a physical injury between the unionized and non-unionized facilities (43% vs. 47%, p = .18).

The mean reported incidents per day during the three-year period was 26.9 (SD = 6.0) and the mean proportion of daily reported incidents involving injuries to workers was .32 (SD = .10). For the facilities included in qualitative analysis **(**Table 3**)**, a range of .04 to 1.3 incidents per staffed bed were reported over the three-year period, while the percentage of incidents involving injury to staff during that time ranged from 4% to 75%. For Facilities 1, 3, 4, and 6, multiple hospitals were represented by the interviewees, so mean values with 95% confidence intervals are presented in Table 3.

For the three-year study period, hospitals reported that for 80% (n = 22,234) of incidents, employees faced no ongoing threat from the WV event and therefore no practice or engineering changes were implemented to mitigate future risk. In 10% (n = 2,797) of incidents, hospitals reported that the incident led to a change in work practice or design. Most frequently, for 3.5% of total incidents (n = 979), this involved improved communication among staff about a patient’s risk for violence.

### Qualitative Findings

Through qualitative analysis, the first author identified several key themes and dimensions of organizational practice relevant to WVIRS implementation. A summary of each facility’s characteristics related to each domain is provided in Table 3 and an overall description is provided below.

#### Organizational Leadership

Participants pointed out that WV reporting sits at the intersection of several organizational functions within hospitals, encompassing employee health, hospital security, risk management, and patient care. Based on each organization’s available resources and approach to regulatory compliance, individuals from varying backgrounds are assigned to the management of the WV compliance operation. Several organizations have approached the problem through the lens of security operations, assigning personnel with either a hospital security or law enforcement background to developing and implementing the WV program. For some larger organizations, leadership have created a specialized role within the security or risk management department to develop and implement the WV prevention and reporting program. There are differences in organizational practice based on available resources as small, private facilities have fewer personnel to work with.

#### Reporting Practices

Interviewees described ways they adapted existing incident reporting platforms or created new tools to collect the required data. Some hospitals added investigation of WV incidents to security staff’s or house supervisors’ workloads. Generally, larger systems built upon their existing incident reporting software to collect data directly from frontline staff who experience violence. This approach requires staff to identify an incident as WV, which flags it for risk managers then routes it to the appropriate personnel for inclusion in WVIRS. The smaller systems reported using more manual approaches, such as paper forms and locally stored spreadsheets, to collect facility-level data before entering it into the WVIRS. These approaches rely on phone calls or verbal reports to managers which initiate the investigation into an incident. All interviewees reported challenges in their attempts to report WV incidents through the WVIRS within the required 72 h. Participants agreed that the 72-h timeline does not permit adequate time for investigation of each incident before submitting the report to CalOSHA.

#### Staff Training

California’s regulation requires that all health care facilities train staff in WV identification and prevention at the time of hiring and with annual refreshers. Additional training in physical management and restraint protocols is required for employees working in high-risk functions such as emergency department, behavioral health, and security personnel. Most interviewees reported that they employ online training modules to provide education to the entire workforce efficiently, with several indicating that they purchased modules from hospital security firms who created content to address the requirements found in California’s new law. The approach to in-person training varied, with some interviewees expressing concern that day-long classes, although often requested by employees, were too resource intensive to be broadly implemented.

Multiple interviewees said that they were challenged by the need to accurately determine who needs training beyond the standard online modules. Leadership from two health systems expressed concerns that nursing staff should generally not be performing physical restraints, so providing this type of in-person training is not necessary, even when staff members state that they would like to have this training. They explained that security or law enforcement should take over in these situations and that nursing or patient care staff who use containment techniques only rarely will not be effective and so may be more likely to injure themselves or their patients.

## Discussion

California’s new WVIRS provides needed insight into the incidence of WV in hospitals. While more serious incidents resulting in hospitalization have previously been reported to CalOSHA, hospitals are now required, for the first time, to track and report all incidents where force was used. The WVIRS has the potential to provide longitudinal data to guide hospitals’ practices in developing interventions to reduce WV towards health care workers.

There are multiple levels of action leading to creation of each hospital’s internal WV records and ultimately to what populates the WVIRS. Prior research has established that, at the level most proximal to the incident, workers’ knowledge of reporting requirements and their belief in the utility of reporting are crucial aspects of their decision to formally document an incident of WV (Gillespie *et al*., 2016; Kvas & Seljak, 2014; Sato *et al*., 2013). For many workers, it seems that these barriers to reporting are not adequately addressed. Researchers investigating hospital-based health care workers’ reporting practices found that 62% had experienced an incident of WV in the past 12 months and that only 12% had documented the occurrence using an electronic reporting system, while 45% reported the incident verbally to a supervisor (Arnetz *et al*., 2015).

At the levels of organization beyond front line health care providers, there are additional barriers and facilitators determining what makes it into the record. Morphet and colleagues (2019) have provided useful insight through description of experiences using Australia’s Victorian Health Incident Management System (VHIMS), an incident tracking system with similarities to California’s WVIRS. In their qualitative work with hospital-based nurse managers and occupational health personnel, researchers found that normalization of WV, technical challenges with utilization of the VHIMS reporting system, inconsistent guidance, and lack of confidence that data would be used to make changes contributed to underreporting of WV incidents through the system. While the researchers’ findings did not link these themes to quantitative outcomes, it is likely that California’s nurse managers and risk managers may confront similar barriers to full and accurate WV reporting.

It is hard to tell from our findings whether those systems relying on workers to directly populate their incident reporting databases are under-capturing to a greater or lesser extent than other approaches. Although workers may be more likely to report verbally, the manager receiving the report may have multiple competing priorities so may not complete the documentation. In our qualitative sample, Facility 1 relied on security personnel to gather information and submit reports, resulting in the most incidents per staffed beds (1.34) and the lowest percentage with an injury (4%). For Facility 6, where a house manager is responsible for investigating and documenting WV incidents, there were the fewest incidents per staffed bed (0.04) with the highest percent involving injury (75%). It may be the case that assigning workers with fewer competing demands on their time results in more thorough documentation of less serious incidents.

Unions’ presence in hospitals did not have a significant impact on the percentage of reported incidents involving injuries to workers. Efforts to enact California’s WV legislation were spearheaded by two of the powerful health care workers’ unions in the state, the California Nurses Association and the Service Employees International Union. Considering these organizations’ leadership on the issue, it seemed relevant to weigh their impact on hospitals’ implementation of the standard. In the absence of strong regulatory guidance, unions have the potential to exert influence over organizational practices that safeguard workers’ safety. Health care worker unions’ significant role in upholding quality and safety standards was identified in recent research from New York State nursing homes where facilities with unionized workforces saw fewer coronavirus-related deaths than non-union facilities (Dean *et al*., 2020). Considering the multiple organizational requirements found in the statute, it is possible that unions have focused their resources on increasing staff training and other violence prevention efforts with more immediately tangible benefits to their members.

### Implications for Practice, Research, and Policy

Hospitals find themselves in a contradictory position when it comes to WV reporting: they want their numbers to reflect their successes in WV prevention and de-escalation, so when few reports appear, it is seemingly cause for celebration. It seems counterintuitive to go looking for a problem. At the same time, epidemiologic studies of WV in health care settings across all types of facilities have demonstrated WV is a pervasive problem with significant consequences for workforce stability and well-being.

It is therefore important for facilities to devote resources to educating workers about WV identification and reporting, and to provide support for the logistical burdens involved in incident reporting. Hospital leaders should develop and deliver training material with knowledge of both the longstanding propensity for WV underreporting and the likely damage it does to workers’ ability to perform their essential roles. Leadership should also recognize that relying solely on health care providers with extensive patient care responsibilities to investigate and document incidents will likely result in incompleteness. Where possible, facilities should allocate additional resources to ensuring reports are accurate and timely and that recommendations for changes in the environment or in practice are solicited from those most impacted.

For researchers, an important contribution of the WVIRS database is that it can provide a longitudinal measure of what interventions have reduced WV occurrences over time. While facilities are required to indicate what steps they took in the aftermath of a reported incident, the vast majority of incidents resulted in no changes to the physical environment or to practice. This is an important area in need of clarification. Facilities in the qualitative sample pointed out that the stringent 72-h timeline did not provide adequate time to fully investigate the precipitants for an incident, so it is unclear whether they are simply choosing to report that no changes were required as a way to complete the form on time, or if the investigation has truly revealed that no changes were necessary. In order to learn from the experience of the state’s hospitals, it will be useful to ask those completing the form to explain how they are answering questions about steps taken to address safety concerns. More broadly, the qualitative findings from this study have identified ways that a small number of California hospitals’ reporting practices vary. Based on these initial findings, it is now possible to consider the impact of organizational practices on a larger scale, potentially through a survey which can be distributed throughout the state. With a more complete picture of the diversity in reporting practices and its impact on WVIRS data, CalOSHA and CDPH can provide additional guidance for reporting facilities and improve consistency.

### Limitations

This study has several limitations. The COREQ checklist for qualitative studies provides a basis for the initial discussion which follows (Tong *et al*., 2007). First, qualitative data collection was curtailed prior to project completion due to the start of the coronavirus pandemic. While it is difficult to anticipate what additional themes or hospital practices could have been identified with additional site visits or interviews, it is possible that more complete descriptions or additional domains for comparison could have emerged. A second limitation is that most facilities were recruited by the CHA, an entity seeking to reduce the burden of regulation for participating facilities. The potential impact of this relationship was noted during interviews and reflections summarized following each interview in an attempt to fully describe how the presence of a CHA representative might have impacted findings. In this study, there were anticipated benefits to using interviewers’ notes instead of recordings, but there are likely to be areas of misinterpretation or incompleteness. Also, because there were only two interviewers interested in broad themes, no formal interview guide was created. In addition, resource constraints allowed for only one data coder which introduces the potential for bias. Acknowledging these limitations, the qualitative findings presented are therefore in some ways preliminary; however, the data collected do provide a strong basis for further investigation and can be used alongside the quantitative results presented.

For the quantitative data, limitations of data collected using the WVIRS have been described by CalOSHA on their website (California Occupational Safety and Health Administration, 2018), and inconsistencies in reporting practices are the subject of this study. Data on staffed beds were missing for several hospitals included in the mixed methods analysis. Sensitivity analysis is included to provide an estimate of the impact of imputations.

## Conclusion

While California’s new WVIRS has generated useful data for more fully describing the epidemiology of this important workplace safety issue, its greater utility has yet to be realized. As a result of California’s new law, hospital-based safety committees are required to discuss WV incidents on a regular basis. These collaborative efforts between workers and managers have the potential to identify important interventions for violence prevention that may be generalizable to other health care settings. It is essential that complete and accurate data collection be prioritized to track outcomes of changes to work practice or design and provide evidence that might lead to wider adoption of innovations. Hospital resources throughout the U.S. are being newly mobilized for WV prevention to comply with the Joint Commission requirements, recent state-level legislation, and in response to grassroots efforts like the one promoted by the American Nurses Association’s #EndNurseAbuse campaign. California’s experience can help inform decision making and improve efficacy of these important initiatives.

## References

[bibr1-15271544221088248] Arnetz J. E. Hamblin L. Ager J. Luborsky M. Upfal M. J. Russell J. Essenmacher L. (2015). Underreporting of workplace violence: comparison of self-report and actual documentation of hospital incidents. Workplace Health & Safety, 63(5), 200–210. 10.1177/2165079915574684 26002854 PMC5006066

[bibr2-15271544221088248] Bensley L. Nelson N. Kaufman J. Silverstein B. Kalat J. Shields J. W. (1997). Injuries due to assaults on psychiatric hospital employees in Washington state. American Journal of Industrial Medicine, 31(1), 92–99. 10.1002/(SICI)1097-0274(199701)31:1 8986260

[bibr3-15271544221088248] Benson A. Secker J. Balfe E. Lipsedge M. Robinson S. Walker J. (2003). Discourses of blame: Accounting for aggression and violence on an acute mental health inpatient unit. Social Science & Medicine (1982), 57(5), 917–926. 10.1016/S0277-9536(02)00460-4 12850116

[bibr4-15271544221088248] Braun V. Clarke V. (2006). Using thematic analysis in psychology. Qualitative Research in Psychology, 3(2), 77–101. 10.1191/1478088706qp063oa

[bibr6-15271544221088248] Byon H. D. Sagherian K. Kim Y. Lipscomb J. Crandall M. Steege L. (2021). Nurses’ experience With type II workplace violence and underreporting during the COVID-19 pandemic. Workplace Health & Safety. Advance online publication. 10.1177/21650799211031233 34344236

[bibr7-15271544221088248] California Occupational Safety and Health Administration (2016). *Violence Prevention in Health Care*. State of California, Department of Industrial Relations. Retrieved from https://www.dir.ca.gov/Title8/3342.html.

[bibr8-15271544221088248] California Occupational Safety & Health Administration (2018). *Workplace Violent Incidents at Hospitals*. State of California, Department of Industrial Relations Retrieved from https://www.dir.ca.gov/dosh/WPVIH_Annual_Reports.html.

[bibr9-15271544221088248] California Office of Statewide Health Planning and Development (OSHPD) (2020). Hospital Quarterly Financial & Utilization Report - Complete Data Set. Retrieved from https://data.chhs.ca.gov/dataset/hospital-quarterly-financial-utilization-report-complete-data-set. 10.1177/2325967120951554 PMC7522844 33029543

[bibr10-15271544221088248] Creswell J. W. Clark V. L. P. (2017). Designing and conducting mixed methods research. Sage publications.

[bibr11-15271544221088248] Dean A. Venkataramani A. Kimmel S. (2020). Mortality rates From COVID-19 Are lower In unionized nursing homes. Health affairs (Project Hope), 39(11), 1993–2001. 10.1377/hlthaff.2020.01011 32910688

[bibr12-15271544221088248] Findorff M. J. McGovern P. M. Wall M. Gerberich S. G. Alexander B. (2004). Risk factors for work related violence in a health care organization. Injury Prevention, 10(5), 296–302. 10.1136/ip.2003.004747 15470011 PMC1730138

[bibr13-15271544221088248] Findorff M. J. McGovern P. M. Wall M. M. Gerberich S. G. (2005). Reporting violence to a health care employer: A cross-sectional study. AAOHN journal : official journal of the American Association of Occupational Health Nurses, 53(9), 399. https://doi-org./10.1177/216507990505300906 16193912

[bibr14-15271544221088248] Forte L. Lanctot N. Geoffrion S. Marchand A. Guay S. (2017). Experiencing violence in a psychiatric setting: generalized hypervigilance and the influence of caring in the fear experienced. Work-a Journal of Prevention Assessment & Rehabilitation, 57(1), 55–67. 10.3233/wor-172540 28506014

[bibr15-15271544221088248] Gacki-Smith J. Juarez A. M. Boyett L. Homeyer C. Robinson L. MacLean S. L. (2009). Violence against nurses working in US emergency departments. JONA: The Journal of Nursing Administration, 39(7/8), 340–349. 10.1097/NNA.0b013e3181ae97db 19641432

[bibr16-15271544221088248] Gillespie G. L. Leming-Lee T. S. Crutcher T. Mattei J. (2016). Chart It to stop It: A quality improvement study to increase the reporting of workplace aggression. Journal of Nursing Care Quality, 31(3), 254–261. 10.1097/NCQ.0000000000000172 26796974

[bibr17-15271544221088248] Jacobsen M. L. F. (2016). *Workplace violence, organizational culture, and registered nurses’ incident reporting patterns in acute hospitals in California* [Ed.D., University of San Francisco]. ProQuest Dissertations & Theses A&I. Ann Arbor.

[bibr18-15271544221088248] The Joint Commission (2021). *New and Revised Workplace Violence Prevention Requirements.* Retrieved from https://www.jointcommission.org/standards/prepublication-standards/new-and-revised-workplace-violence-prevention-requirements.

[bibr20-15271544221088248] Kim H. Sefcik J. S. Bradway C. (2017). Characteristics of qualitative descriptive studies: A systematic review. Research in Nursing & Health, 40(1), 23–42. 10.1002/nur.21768 27686751 PMC5225027

[bibr21-15271544221088248] Kvas A. Seljak J. (2014). Unreported workplace violence in nursing. International Nursing Review, 61(3), 344–351. 10.1111/inr.12106 24847955

[bibr22-15271544221088248] Liu J. Gan Y. Jiang H. Li L. Dwyer R. Lu K. Yan S. Sampson O. Xu H. Wang C. Zhu Y. Chang Y. Yang Y. Yang T. Chen Y. Song F. Lu Z. (2019). Prevalence of workplace violence against healthcare workers: A systematic review and meta-analysis. Occupational and Environmental Medicine, 76(12), 927–937. 10.1136/oemed-2019-105849 31611310

[bibr23-15271544221088248] McGuire S. S. Gazley B. Majerus A. C. Mullan A. F. Clements C. M. (2021). 69 Impact of the COVID-19 pandemic on workplace violence at an academic emergency department. Annals of Emergency Medicine, 78(2), S33–S34. 10.1016/j.annemergmed.2021.07.071 PMC8457914 34602329

[bibr24-15271544221088248] Meyer J. W. Rowan B. (1977). Institutionalized organizations: formal structure as myth and ceremony. American Journal of Sociology, 83(2), 340–363. 10.1086/226550

[bibr25-15271544221088248] Morphet J. Griffiths D. Innes K. (2019). The trouble with reporting and utilization of workplace violence data in health care. Journal of Nursing Management, 27(3), 592–598. 10.1111/jonm.12717 30223311

[bibr26-15271544221088248] Occupational Safety and Health Administration (2020). *Recordkeeping and Reporting requirements*. Retrieved from: https://www.osha.gov/recordkeeping/entryfaq.html.

[bibr27-15271544221088248] Odes R. Hong O. Harrison R. Chapman S. (2020). Factors associated with physical injury or police involvement during incidents of workplace violence in hospitals: findings from the first year of california's new standard. American Journal of Industrial Medicine, 63(6), 543–549. 10.1002/ajim.23103 32166835

[bibr28-15271544221088248] Palinkas L. A. Aarons G. A. Horwitz S. Chamberlain P. Hurlburt M. Landsverk J. (2011). Mixed method designs in implementation research. Administration and Policy in Mental Health and Mental Health Services Research, 38(1), 44–53. 10.1007/s10488-010-0314-z 20967495 PMC3025112

[bibr29-15271544221088248] Peek-Asa C. Runyan C. W. Zwerling C. (2001). The role of surveillance and evaluation research in the reduction of violence against workers. American Journal of Preventive Medicine, 20(2), 141–148. 10.1016/S0749-3797(00)00290-7 11165457

[bibr31-15271544221088248] Sato K. Wakabayashi T. Kiyoshi-Teo H. Fukahori H. (2013). Factors associated with nurses’ reporting of patients’ aggressive behavior: A cross-sectional survey. International Journal of Nursing Studies, 50(10), 1368–1376. 10.1016/j.ijnurstu.2012.12.011 23305760

[bibr32-15271544221088248] State of California Legislative Counsel (2014). *Senate Rules Committee: Bill Analysis SB 1299*. Retrieved from http://leginfo.ca.gov/pub/13-14/bill/sen/sb_1251-1300/sb_1299_cfa_20140527_170449_sen_floor.html.

[bibr33-15271544221088248] Tong A. Sainsbury P. Craig J. (2007). Consolidated criteria for reporting qualitative research (COREQ): A 32-item checklist for interviews and focus groups. International Journal for Quality in Health Care, 19(6), 349–357. 10.1093/intqhc/mzm042 17872937

[bibr34-15271544221088248] Ulrich B. Barden C. Cassidy L. Varn-Davis N. (2019). Critical care nurse work environments 2018: findings and implications. Critical Care Nurse, 39(2), 67–84. 10.4037/ccn2019605 30728131

[bibr35-15271544221088248] United States Congress (2021). *H.R.1195 - Workplace Violence Prevention for Health Care and Social Service Workers Act*. Retrieved from https://www.congress.gov/bill/117th-congress/house-bill/1195/text.

[bibr36-15271544221088248] Vaismoradi M. Turunen H. Bondas T. (2013). Content analysis and thematic analysis: implications for conducting a qualitative descriptive study. Nursing & Health Sciences, 15(3), 398–405. 10.1111/nhs.12048 23480423

